# Long-Acting Injectable Cabotegravir Use and Persistence Over 2 Years

**DOI:** 10.1001/jamanetworkopen.2026.20699

**Published:** 2026-06-26

**Authors:** Shi Hao Ernest Koh, Wenting Huang, Eric W. Hall, Jeb Jones, Xiao Zang, Courtney R. Yarbrough, Edwin E. Corbin-Gutierrez, Patrick S. Sullivan, Aaron J. Siegler

**Affiliations:** 1 Department of Epidemiology, Rollins School of Public Health, Emory University, Atlanta, Georgia; 2 OHSU-PSU School of Public Health, Oregon Health & Science University, Portland; 3 Division of Health Policy and Management, School of Public Health, University of Minnesota, Minneapolis; 4 Department of Health Policy and Management, Emory University, Atlanta, Georgia; 5 National Alliance of State and Territorial AIDS Directors, Washington, DC

## Abstract

**Importance:**

Long-acting injectable (LAI) pre-exposure prophylaxis (PrEP) has superior efficacy vs oral PrEP, and higher LAI–cabotegravir (CAB) persistence may optimize its HIV prevention impact.

**Objective:**

To characterize individuals using LAI-CAB compared with oral PrEP, to describe changes in national LAI-CAB vs oral PrEP use over time, and to examine longitudinal persistence among LAI-CAB users.

**Design, Setting, and Participants:**

This retrospective cohort study used pharmacy and medical claims from a database that covered a majority of all claims in the US from 2022 to 2024. Participants included persons with 1 or more claim for any PrEP drugs (oral or injectable).

**Exposure:**

PrEP use during the study period.

**Main Outcomes and Measures:**

Study outcomes were LAI-CAB use and persistence in care. LAI-CAB use was defined as at least 1 claim, and LAI-CAB yearly persistence was defined as at least 2 LAI-CAB claims for each of 2 biannual periods. Yearly persistence in any type of PrEP (PrEP persistence) was defined as at least 2 claims for PrEP medications (oral or injectable) for each of 2 biannual periods among LAI-CAB users. Multivariable logistic regression models were used to assess variables associated with LAI-CAB use compared with oral PrEP.

**Results:**

From 2022 to 2024, there were 24 194 LAI-CAB users, representing 3% of all 781 040 PrEP users. LAI-CAB users were young (mean [SD] age, 37.1 [11.4] years) and predominantly male (20 642 users [85%]); 656 (3%) were Asian, 4531 (19%) were Black, 4380 (19%) were Hispanic, 13 696 (58%) were White, and 414 (2%) were of other races and ethnicities. Most had commercial insurance (16 334 users [68%]) and had no copayment (13 507 users [82%]). LAI-CAB users were more likely than oral PrEP users to be covered by Medicaid (6256 of 24 194 users [26%] vs 108 589 of 770 833 users [14%]). In the most recent evaluated period (July to December 2024), LAI-CAB accounted for 4% (16 557 of 390 816 users) of PrEP users. Among users with sufficient data, LAI-CAB persistence was 50% (6020 of 12 118 users) at 1 year and 23% (785 of 3381 users) at 2 years. Overall PrEP persistence among LAI-CAB users, including transitions to oral PrEP, was 57% (6879 of 12 118 users) at 1 year and 30% (1019 of 3381 users) at 2 years. A greater proportion of female users initiated LAI-CAB (3551 users [15%]) than oral PrEP (83 274 users [11%]), but 2-year LAI-CAB persistence was lower among female users (61 users [13%]) than among male users (724 users [25%]).

**Conclusions and Relevance:**

In this national prescription claims cohort study, LAI-CAB use was a small share of overall PrEP use from 2022 to 2024. Approximately one-half of users were persistent in LAI-CAB or in PrEP at year 1, declining to about one-quarter and one-third, respectively, by year 2. The implementation of more effective new PrEP modalities alone is unlikely to substantially alter PrEP use in the US. Instead, structural supports and behavioral interventions are needed to facilitate scale-up of new, highly efficacious modalities.

## Key Points

**Question:**

What are the levels of long-acting injectable-cabotegravir (LAI-CAB) uptake and persistence in the US since Food and Drug Administration approval in December 2021?

**Findings:**

In this cohort study of national prescription claims from 2022 to 2024, there were 24 194 LAI-CAB users, representing 3% of 781 040 US pre-exposure prophylaxis (PrEP) users. Persistence in LAI-CAB care was 51% in the first year and 23% in the second year; persistence in PrEP care among LAI-CAB users, including oral modalities, was 57% in the first year and 30% in the second year.

**Meaning:**

These findings suggest that LAI-CAB had modest uptake and persistence in care and that structural interventions to facilitate access to highly effective long-acting PrEP modalities are needed.

## Teaser

This cohort study characterizes individuals using long-acting injectable cabotegravir compared with oral pre-exposure prophylaxis, describes changes in national use over time, and examines longitudinal persistence among users.

## Introduction

In the US, HIV remains a substantial burden, with an estimated more than 38 000 new diagnoses in 2024.^1^ Long-acting injectable (LAI) cabotegravir (CAB) was approved by the US Food and Drug Administration in December 2021 as the first LAI pre-exposure prophylaxis (LAI-PrEP) with injections once every 2 months, offering an alternative to daily oral PrEP.^2^ In June 2025, lenacapavir was approved as a twice-a-year LAI-PrEP.^3^ LAI-CAB has been hailed as a great advancement in HIV prevention and is expected to have a major impact on the HIV epidemic.^4,5,6,7,8^ Clinical trials found that compared with oral PrEP (emtricitabine [FTC]–tenofovir disoproxil fumarate [TDF]), LAI-CAB was associated with 66% reduced risk of HIV infection among men who have sex with men and transgender women,^9^ and 88% reduced risk among cisgender women.^10^ Across studies of hypothetical PrEP modalities among PrEP-eligible people, 52% to 81% of participants preferred LAI-CAB over oral PrEP.^11,12,13,14,15^ However, by the end of 2023, LAI-CAB had only modest uptake, accounting for just 2.5% of US PrEP users.^16^

The discrepancy between LAI-CAB interest and uptake suggests a translational gap. A systematic review of LAI-CAB preferences indicated variation by sociodemographic and behavioral characteristics.^17^ Many individual-level behavioral factors are dynamic and may change over time, although none of these is sufficient to solely account for the gap between anticipated and actual LAI-CAB use. Structural challenges in LAI-CAB administration include medication acquisition, logistics, and insurance requirements, complicating its rollout. Medication acquisition models, including buy-and-bill and pharmacy distribution, may pose operational challenges that strain clinic infrastructure and administrative capacity.^18^ In addition, complex insurance processes, including prior authorization, can complicate LAI-CAB administrative burden.^18^ Together, these barriers have impeded the scale-up of LAI-CAB.

Although national data on the overall number of LAI-CAB users have been published,^16^ there is a need to provide additional context in the form of use over time and user characteristics to understand how LAI-CAB has come to scale in this complex provision environment. LAI-CAB was the sole LAI-PrEP option from 2022 to 2024, and limited data exist regarding how individuals are engaged in care over longer use periods. Understanding LAI-CAB uptake and persistence has the potential to inform implementation strategies for other LAI-PrEP modalities. We sought to (1) characterize individuals using LAI-CAB compared with oral PrEP in the US from 2022 to 2024, (2) describe changes in national LAI-CAB vs oral PrEP use over time, and (3) examine longitudinal persistence among LAI-CAB users.

## Methods

### Study Design

This retrospective cohort study used deidentified, deduplicated commercial medical and pharmacy claims data in the US from Symphony Health (ICON plc Company), PatientSource, January 1, 2022, to December 31, 2024, which captures most PrEP prescriptions in the US but is limited as it excludes closed health care systems (Kaiser Permanente and Veterans Affairs). We characterized LAI-CAB uptake compared with oral PrEP use descriptively and evaluated persistence among LAI-CAB users. For earlier studies of oral PrEP in the context of a single available PrEP modality, persistence was often defined as the continued use of the same regimen.^19^ With the expansion of PrEP options, more-nuanced persistence analyses are necessary because discontinuation of a specific modality may reflect a modality switch rather than cessation. We distinguished between LAI-CAB persistence and persistence in any type of PrEP (PrEP persistence), the latter allowing for modality switches to oral PrEP.

### Ethical and Reporting Considerations

This study analyzed deidentified data and is not human participants research defined by federal regulations. Investigators completed a worksheet provided by the Emory University institutional review board, documenting that institutional review board review was not required. For this report, we followed Strengthening the Reporting of Observational Studies in Epidemiology (STROBE) reporting guidelines.

### Algorithm and Data Extraction

PrEP claims were identified and abstracted from the national health claims data using an algorithm we developed.^20^ We included claims for approved PrEP medications (FTC-TDF, FTC–tenofovir alafenamide [TAF], and CAB) and excluded those for HIV treatment and postexposure prophylaxis based on dual-drug and triple-drug antiretroviral regimen data and postexposure prophylaxis or HIV diagnosis data.

### Variables of Interest

Sociodemographic data, including sex, race and ethnicity (categorized as Asian, Black or African American, Hispanic, White, and any other race not otherwise specified), age, state of residence, insurance payer type, and copayment (given in US dollars throughout), were extracted from patients’ first recorded PrEP claim during the study period. Data on race and ethnicity are included in this study to characterize the study population and provide contextual data to aid in interpretation of the findings.

### Interval Classification of PrEP Usage

To assess uptake of oral PrEP and LAI-CAB from 2022 to 2024, we categorized claims dates into biannual periods; for instance, if a user started January 1, their first period would be through June 30 and their second period through December 31. Because oral and LAI-CAB have different periods between prescriptions, biannual periods were selected to allow for meaningful comparisons across formulations, in a manner that will also be applicable in the future for biannual injectable lenacapavir PrEP. A patient was considered an oral PrEP user in each biannual period if they had at least 1 claim of TDF-FTC or TAF-FTC, and a LAI-CAB user if they had at least 1 claim for CAB. Patients who switched between formulations were counted in both categories if they had claims for both formulations during the same period. We assessed LAI-CAB uptake across US states and territories using heat maps showing the cumulative number of PrEP users and the PrEP-to-need ratio,^21^ operationalized as the number of LAI-CAB users per 100 new HIV diagnoses from 2022 to 2024. Rates were calculated using new HIV diagnosis data from AIDSVu for 2022 and 2023^22^ because 2024 data were not yet available.

### LAI-CAB and PrEP Persistence

For LAI-CAB persistence, we assessed users’ medication claims history from the time they initiated LAI-CAB to the end of 2024. Persistence for a year was defined as a patient having at least 2 LAI-CAB doses in each biannual period. Using 6-monthly windows allows capturing at least 2 injections per interval without prematurely classifying a person as not persistent given scheduling variations that reflect the realities of ongoing engagement in care. These criteria are intended as minimum indicators of patient persistence in PrEP care. We assessed PrEP persistence with a similar framework, defining it as having at least 2 claims for any PrEP medications within each biannual period, thus accounting for modality switch. Users with at least 12 months and 24 months of follow-up from initiation to end of study period were included in 1-year and 2-year persistence analyses, respectively. Persistence was stratified by sociodemographic and health insurance variables. Sensitivity analyses were conducted using alternative triannual and annual definitions (eTables 1 and 2 in Supplement 1).

### Statistical Analysis

Patient characteristics and trends in PrEP uptake and persistence were summarized descriptively. We used multivariable logistic regression models as exploratory, hypothesis-generating analyses to assess variables associated with LAI-CAB use compared with oral PrEP. The dependent variable was ever LAI-CAB use, defined as having used LAI-CAB at any point. Covariates were selected on the basis of their conceptual relevance to PrEP use, including sociodemographic and insurance-related variables. Missing race and ethnicity data (48% of race and ethnicity data were missing) were imputed using hot deck imputation,^23^ with donor pools defined using variables selected according to availability, such as sex, payer type, patient state of residence, and age category. We also used the missing indicator method for sensitivity analyses (eTables 3-5 in Supplement 1).^24^ Other covariates with missing data were excluded from the regression analyses given substantially lower rates of missingness; 45 010 patients were excluded for this reason. Collinearity was evaluated, and model fit was assessed using Hosmer-Lemeshow goodness-of-fit tests. Model discrimination was evaluated using C statistics. Statistical significance was set at 2-sided *P* < .05. Analyses were conducted using SAS statistical software version 9.4 (SAS Institute), and figures were created using R statistical software version 4.5.1 (R Project for Statistical Computing).

## Results

### Demographic Characteristics

There were 781 040 PrEP users in the US from 2022 to 2024; 770 833 used oral PrEP at least once, and 24 194 (3%) used LAI-CAB at least once. LAI-CAB users were young (mean [SD] age, 37.1 [11.4] years) and predominantly male (20 642 users [85%]); 656 (3%) were Asian, 4531 (19%) were Black, 4380 (19%) were Hispanic, 13 696 (58%) were White, and 414 (2%) were other races. Most had commercial insurance (16 334 users [68%]) and had no copayment (13 507 users [82%]). User characteristics are shown in Table 1. Compared with oral PrEP users, a higher proportion of LAI-CAB users were aged 31 to 40 years (8828 users [36%] vs 253 274 users [33%]) or 41 to 50 years (4281 users [18%] vs 118 474 users [15%]), were female (3551 users [15%] vs 83 274 users [11%]), were Black (4531 users [19%] vs 120 924 users [16%]), were enrolled in Medicaid (6256 users [26%] vs 108 589 users [14%]), and had a copayment exceeding $1000 (705 users [4%] vs 6841 users [1%]). Compared with LAI-CAB users, a higher proportion of oral PrEP users were aged 19 to 30 years (279 749 users [36%] vs 7531 users [31%]), were male (687 541 users [89%] vs 20 642 users [85%]), were White (467 041 users [62%] vs 13 696 users [58%]), were enrolled in assistance programs (eg, manufacturer-sponsored and government programs; 118 534 users [15%] vs 509 users [2%]), and had a copayment of $11 to $100 (69 120 users [9%] vs 1040 users [6%]).

**Table 1.** Sociodemographic Characteristics of PrEP Users and Multivariable Logistic Regression of LAI-CAB Use

Characteristic	Users, No. (%)^a^			Ever LAI-CAB vs oral PrEP only, aOR (95% CI) (n = 736 030)
	PrEP overall (N = 781 040)	Oral PrEP only (n = 770 833)	LAI-CAB (n = 24 194)	
Age, mean (SD), y	36.4 (11.9)	36.4 (11.9)	37.1 (11.4)	NA
≤18	6983 (1)	6854 (1)	175 (1)	0.70 (0.60-0.81)
19-30	283 110 (36)	279 749 (36)	7531 (31)	0.83 (0.80-0.86)
31-40	256 825 (33)	253 274 (33)	8828 (36)	1 [Reference]
41-50	120 211 (15)	118 474 (15)	4281 (18)	1.00 (0.96-1.05)
>50	113 911 (15)	112 482 (15)	3379 (14)	0.81 (0.78-0.85)
Sex at birth^b^				
Male	695 388 (89)	687 541 (89)	20 642 (85)	1 [Reference]
Female	85 633 (11)	83 274 (11)	3551 (15)	1.10 (1.06-1.15)
Race and ethnicity^c^				
Asian	24 852 (3)	24 602 (3)	656 (3)	0.90 (0.83-1.11)
Black or African American	122 985 (16)	120 924 (16)	4531 (19)	1.21 (1.17-1.26)
Hispanic	128 564 (17)	126 776 (17)	4380 (19)	1.16 (1.12-1.21)
White	472 671 (62)	467 041 (62)	13 696 (58)	1 [Reference]
Other^d^	13 664 (2)	13 483 (2)	414 (2)	1.00 (0.89-1.11)
US Census region^b^				
Northeast	176 024 (23)	173 405 (23)	6003 (25)	1.15 (1.11-1.19)
Midwest	123 936 (16)	122 512 (16)	3640 (15)	1.01 (0.97-1.06)
South	288 313 (37)	284 997 (37)	8347 (35)	1 [Reference]
West	174 382 (22)	171 867 (22)	5610 (23)	1.04 (1.00-1.08)
Territories	1864 (<1)	1836 (<1)	57 (<1)	1.08 (0.80-1.46)
Payer type^b^				
Commercial	520 012 (67)	513 746 (67)	16 334 (68)	1 [Reference]
Government	14 (<1)	3 (<1)	19 (<1)	NA
Medicare	21 590 (3)	21 220 (3)	851 (4)	1.68 (1.55-1.82)
Medicaid	111 929 (14)	108 589 (14)	6256 (26)	2.50 (2.41-2.59)
Assistance programs	118 658 (15)	118 534 (15)	509 (2)	0.68 (0.65-0.72)
Cash	3369 (<1)	3278 (<1)	207 (1)	0.49 (0.31-0.80)
Copayment amount, US$^b^				
0	625 519 (83)	619 959 (83)	13 507 (82)	1 [Reference]
1-10	33 243 (4)	33 024 (4)	477 (3)	0.75 (0.70-0.81)
11-100	69 395 (9)	69 120 (9)	1040 (6)	1.04 (0.99-1.10)
101-500	14 991 (2)	14 836 (2)	471 (3)	1.43 (1.30-1.57)
501-1000	6246 (1)	6184 (1)	181 (1)	1.44 (1.25-1.66)
>1000	7089 (1)	6841 (1)	705 (4)	2.61 (2.35-2.90)

Abbreviations: aOR, adjusted odds ratio; LAI-CAB, long-acting injectable cabotegravir; NA, not applicable; PrEP, pre-exposure prophylaxis.

^a^ Values from oral PrEP and LAI-CAB users columns do not add up to PrEP users column because of persons who used more than 1 type of PrEP medication from 2022 to 2024.

^b^ Values do not add up because of missing information for sex (19 users [<1%]), US Census region (16 521 users [2%]), payer type (5468 users [<1%]), and copayment amount (24 557 users [3%]).

^c^ Missing race and ethnicity data (373 989 users [48%]) were imputed using hot deck imputation method. After imputation, race and ethnicity were missing for 18 304 users (2%).

^d^ Other race is any other race not otherwise specified.

### Multivariable Logistic Regression: Variables Associated With LAI-CAB Use

Compared with oral PrEP use, LAI-CAB use was associated with female sex (adjusted odds ratio [aOR], 1.10 [95% CI, 1.06-1.15]; reference, male sex); Black race (aOR, 1.21 [95% CI, 1.17-1.26]) and Hispanic ethnicity (aOR, 1.16 [95% CI, 1.12-1.21]; reference, White race); residence in the Northeast (aOR, 1.15 [95% CI, 1.11-1.19]; reference, South); younger (≤18 years, aOR, 0.70 [95% CI, 0.60-0.81]; 19-30 years, aOR, 0.83 [95% CI, 0.80-0.86]) and older (>50 years, aOR, 0.81 [95% CI, 0.78-0.85]) age (reference, 31-40 years); all other payer types (Medicare, aOR, 1.68 [95% CI, 1.55-1.82]; Medicaid, aOR, 2.50 [95% CI, 2.41-2.59]; assistance program, aOR, 0.68 [95% CI, 0.65-0.72]; and cash, aOR, 0.49 [95% CI, 0.31-0.80]; reference, commercial); and copayment of $1 to $10 (aOR, 0.75 [95% CI, 0.70-0.81]), $101 to $500 (aOR, 1.43 [95% CI, 1.30-1.57]), $501 to $1000 (aOR, 1.44 [95% CI, 1.25-1.66]), or more than $1000 (aOR, 2.61 [95% CI, 2.35-2.90; reference, $0]). All aORs are shown in Table 1, and unadjusted ORs are shown in eTable 6 in Supplement 1. Model discrimination was modest (C = 0.62), and Hosmer-Lemeshow test indicated suboptimal fit (χ^2^ = 36.6; *P* < .001) and no evidence of collinearity.

### Trends in LAI-CAB Uptake

**Figure 1.**
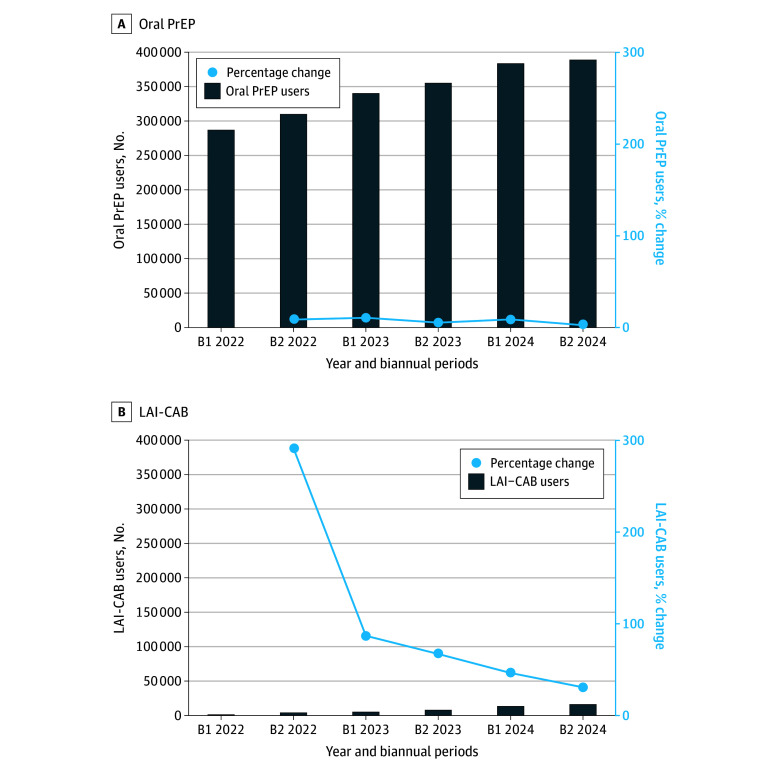
Bar and Line Graphs of Use Trends of Oral Pre-Exposure Prophylaxis (PrEP) and Long-Acting Injectable Cabotegravir (LAI-CAB) Over Time Panel A shows the overall growth of oral PrEP use, from 287 773 users in the first biannual period (B1) of 2022 to 390 816 users in the second biannual period (B2) of 2024, with percentage growth ranging between 1.7% to 9.8% per biannual period. Panel B shows the overall growth of LAI-CAB use, from 704 users in B1 2022 to 16 557 users in B2 2024, with percentage growth ranging between 30.9% to 291.9% per period.

**Figure 2.**
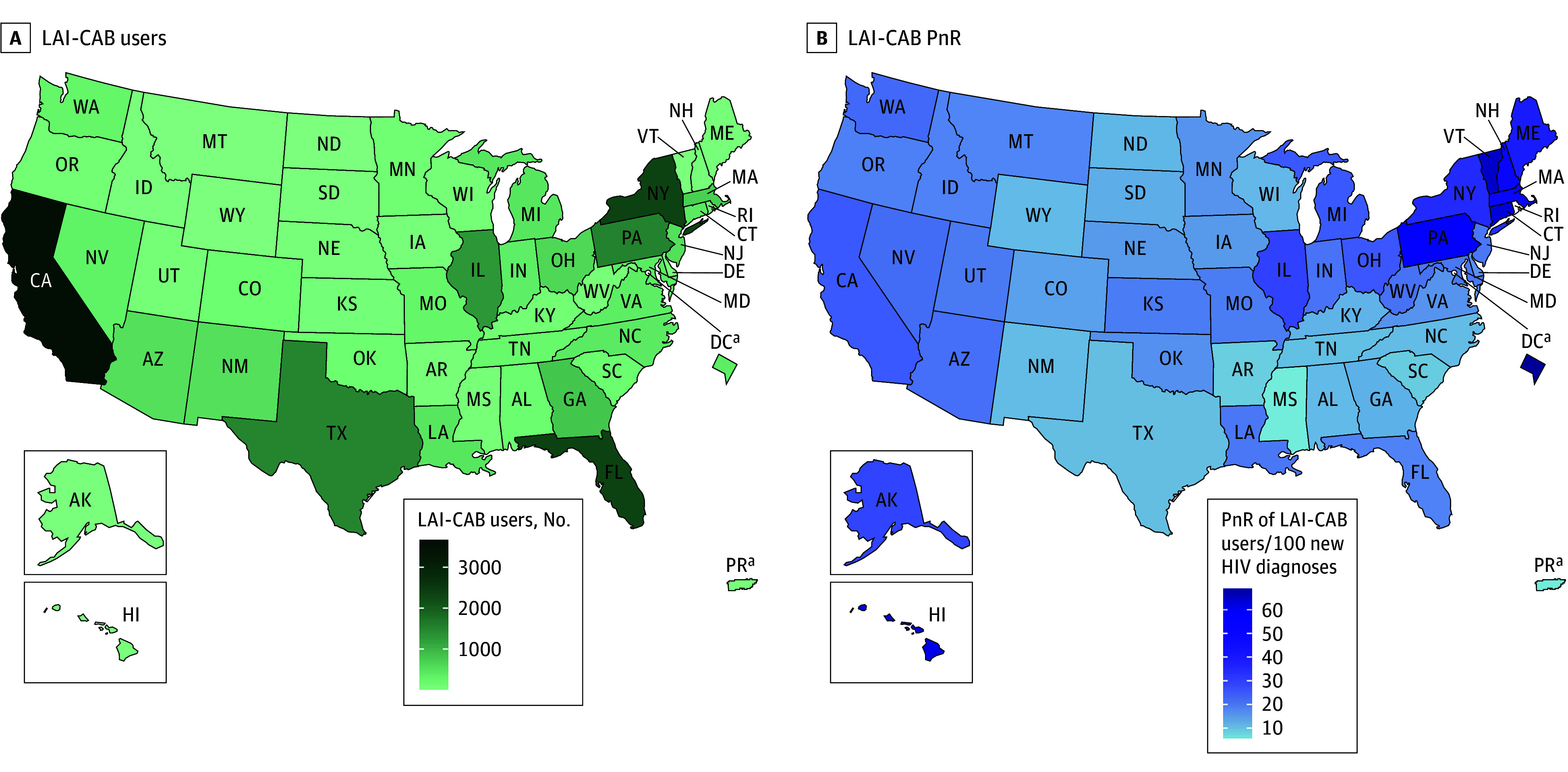
Maps of Cumulative Distribution of Long-Acting Injectable Cabotegravir (LAI-CAB) Users and Pre-Exposure Prophylaxis (PrEP)-to-Need Ratio (PnR) Across US States and Territories Panel A shows the cumulative number of LAI-CAB users (n = 23 657 with n = 537 missing state information), and panel B shows the LAI-CAB PnR across US states, including the District of Columbia (DC) and Puerto Rico (PR). PnR was calculated as the cumulative number of LAI-CAB users from 2022 to 2024 divided by the cumulative number of new HIV diagnoses from 2022 and 2023 (2024 data were not available), expressed per 100 new HIV diagnoses. Higher counts or rates are represented by darker shades on the map. ^a^Not drawn to scale.

From 2022 to 2024, the number of oral PrEP users per biannual period increased from 287 773 to 390 816 (Figure 1A), with an average biannual increase of 20 608 users, or 7.2% (range, 6349 users [1.7%] to 30 387 users [9.8%]). The number of LAI-CAB users increased from 704 to 16 557 (Figure 1B), with an average biannual increase of 3170 users, or 104.7% (range, 2055 users [30.9%] to 4016 users [291.9%]). California had the highest cumulative number of LAI-CAB users (3692 users) (Figure 2A), and the District of Columbia had the highest cumulative PrEP-to-need ratio (70 users per 100 new HIV diagnoses) (Figure 2B). The PrEP-to-need ratio was lower in the US Southeast, including Mississippi (5 users per 100 new HIV diagnoses), Arkansas (9 users per 100 new HIV diagnoses), and South Carolina (9 users per 100 new HIV diagnoses).

### LAI-CAB and PrEP Persistence

There were 12 118 LAI-CAB users with at least 1 year of follow-up data (Table 2); 50% (6020 of 12 118 users) of users were persistent in LAI-CAB during their first year of care. Fewer persons aged 18 years or younger were persistent (25 users [29%]) than those aged 51 years and older (993 users [59%]) in the first year of care. Approximately one-half of male users (5456 users [52%]) and about one-third of female users (564 users [34%]) were persistent. LAI-CAB persistence was comparable across racial and ethnic groups. More than one-half of users with commercial insurance (4434 users [53%]), Medicare (211 users [52%]), government insurance (8 users [57%]), or enrolled in assistance programs (45 users [63%]) were persistent, whereas less than one-half of users enrolled in Medicaid (1302 users [41%]) or paying with cash (15 users [39%]) were persistent. Across increasing copayment amounts, LAI-CAB persistence levels were similar. Among LAI-CAB users, when also considering transitions to oral PrEP, overall PrEP persistence was 57% (6879 of 12 118 users), and 1% to 10% higher than LAI-CAB persistence across groups.

**Table 2.** LAI-CAB Persistence (Assessed Biannually) and PrEP Persistence Stratified by Sociodemographic Variables Among Persons With at Least 1 Year of Follow-Up and at Least 2 Years of Follow-Up^a^

Variables	Participants, No. (%)									
	1-y Follow-up (n = 12 118)			2-y Follow-up (n = 3381)						
	LAI-CAB initiation	LAI-CAB persistence, year 1	PrEP persistence, year 1	LAI-CAB initiation	LAI-CAB persistence			PrEP persistence		
					Year 1	Year 2^b^	Year 1-2^c^	Year 1	Year 2^b^	Year 1-2^c^
Overall	12 118	6020 (50)	6879 (57)	3381	1581 (47)	899 (27)	785 (23)	1875 (55)	1196 (35)	1019 (30)
Age, y										
≤18	87	25 (29)	26 (30)	35	11 (31)^d^	4 (11)^d^	3 (9)^d^	12 (34)	5 (14)^d^	4 (11)^d^
19-30	3854	1724 (45)	1966 (51)	1092	462 (42)	232 (21)	199 (18)	556 (51)	307 (28)	254 (23)
31-40	4358	2177 (50)	2499 (57)	1150	550 (48)	307 (27)	261 (23)	643 (56)	416 (36)	349 (30)
41-50	2123	1101 (52)	1278 (60)	580	258 (44)	162 (28)	140 (24)	318 (55)	224 (39)	184 (32)
>50	1696	993 (59)	1110 (65)	524	300 (57)	194 (37)	182 (35)	346 (66)	244 (47)	228 (44)
Sex^e^										
Male	10 462	5456 (52)	6268 (60)	2919	1436 (49)	832 (29)	724 (25)	1717 (59)	1120 (38)	951 (33)
Female	1655	564 (34)	611 (37)	462	145 (31)	67 (15)	61 (13)	158 (34)	76 (16)	68 (15)
Race and ethnicity^f^										
Asian	334	164 (49)	192 (57)	96	48 (50)	21 (22)	19 (20)	56 (58)	31 (32)	26 (27)
Black or African American	2292	1057 (46)	1188 (52)	619	274 (44)	155 (25)	132 (21)	308 (50)	198 (32)	160 (26)
Hispanic	2245	1114 (50)	1293 (58)	661	321 (49)	166 (25)	148 (22)	382 (58)	234 (35)	199 (30)
White	6749	3466 (51)	3961 (59)	1872	894 (48)	533 (28)	465 (25)	1081 (58)	700 (37)	607 (32)
Other^g^	219	107 (49)	118 (54)	70	23 (33)	13 (19)	12 (17)	27 (39)	22 (31)	18 (26)
Payer type^e^										
Commercial	8402	4434 (53)	5082 (60)	2271	1102 (49)	640 (28)	552 (24)	1331 (59)	878 (39)	741 (33)
Government	14	8 (57)^d^	9 (64)^d^	1	0	0	0	0	0	0
Medicare	408	211 (52)	243 (60)	133	66 (50)	45 (34)	42 (32)	79 (59)	53 (40)	48 (36)
Medicaid	3174	1302 (41)	1469 (46)	955	402 (42)	204 (21)	182 (19)	450 (47)	251 (26)	218 (23)
Assistance programs	71	45 (63)	52 (73)	14	7 (50)^d^	6 (43)^d^	6 (43)^d^	10 (71)^d^	9 (64)^d^	9 (64)
Cash	38	15 (39)	17 (45)	2	1 (50)^d^	1 (50)^d^	1 (50)^d^	1 (50)^d^	2 (100)^d^	1 (50)^d^
Copayment amount, US$^e^										
0	6612	3322 (50)	3795 (57)	1856	885 (48)	483 (26)	439 (24)	1036 (56)	634 (34)	560 (30)
1-10	281	109 (39)	128 (46)	130	41 (32)	30 (23)	25 (19)	51 (39)	35 (27)	28 (22)
11-100	496	280 (56)	319 (64)	148	65 (44)	46 (31)	40 (27)	87 (59)	60 (41)	54 (36)
101-500	239	137 (57)	161 (67)	96	46 (48)	43 (45)	33 (34)	60 (63)	52 (54)	41 (43)
501-1000	77	48 (62)	53 (69)	28	16 (57)	13 (46)	11 (39)^d^	19 (68)	17 (61)	13 (46)
>1000	249	142 (57)	160 (64)	83	36 (43)	24 (29)	21 (25)	44 (53)	31 (37)	26 (31)

Abbreviations: LAI-CAB, long-acting injectable cabotegravir; PrEP, pre-exposure prophylaxis.

^a^ LAI-CAB and PrEP persistence are defined in the Methods The denominators (n) of the respective subgroups are shown in the “Initiation” columns and percentages represent the proportion of users who were persistent.

^b^ Year 2 persistence was assessed regardless of persistence status in year 1.

^c^ Refers to persistence in all 4 biannual periods.

^d^ Percentages generated from a numerator of less than 12 are considered unstable and should be interpreted with caution (adapted from AIDSVu^22^).

^e^ Values do not add up due to missing information (sex, 1 user; race and ethnicity, 279 users; payer type, 11 users, and copayment, 4164 users).

^f^ Missing data for race and ethnicity were hot deck imputed.

^g^ Other race is any other race not otherwise specified.

**Figure 3.**
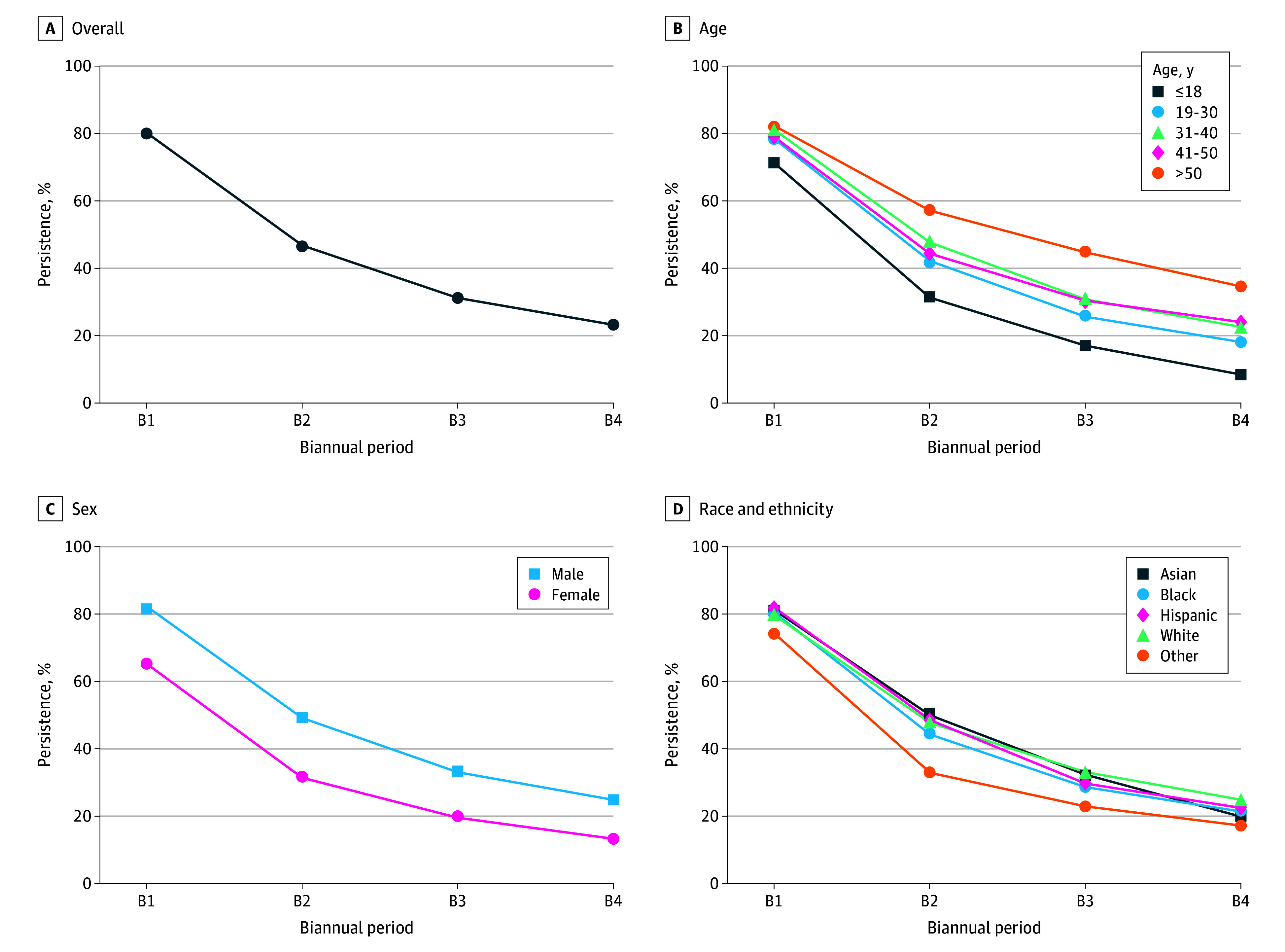
Line Graphs of Biannual Percentage Cumulative Long-Acting Injectable Cabotegravir (LAI-CAB) Persistence Among Users With at Least 2 Years of Follow-Up Data Among users with at least 2 years of follow-up (n = 3381), percentage cumulative persistence in LAI-CAB was assessed across 4 biannual periods. B2 corresponds to year 1 LAI-CAB persistence, and B4 corresponds to year 1 to 2 LAI-CAB persistence, as reported in Table 2. LAI-CAB persistence was stratified across age, sex, and race and ethnicity. Other race includes any other race not otherwise specified.

Of the 12 118 LAI-CAB users, 3381 had at least 2 years of follow-up data. Across the 2-year period, 23% of users (785 of 3381 users) were persistent in LAI-CAB care. Fewer persons aged 18 years or younger were persistent (3 users [9%]) than those aged 51 years or older (182 users [35%]). About 1 in 4 male (724 users [25%]) and 1 in 8 female (61 users [13%]) users were persistent in LAI-CAB over the period. LAI-CAB persistence across racial and ethnic groups was comparable. Higher proportions of users enrolled in Medicare (42 users [32%]) were persistent than those enrolled in commercial insurance (552 users [24%]) or Medicaid (182 users [19%]). Across increasing copayment amounts, LAI-CAB persistence was similar. When considering transitions to oral PrEP for LAI-CAB users, PrEP persistence was 30% (1019 of 3381 users), and 2% to 11% higher than LAI-CAB persistence across groups. Across our persistence analyses, findings were consistent in sensitivity analyses that varied the assessment periods (triannual and annual) and definitions regarding the threshold to classify a user as persistent (eTables 1 and 2 in Supplement 1). Across the 4 biannual periods, cumulative LAI-CAB persistence declined, with a consistent downward trend across all assessed subgroups (Figure 3), indicating that for all user types more current LAI-CAB users cease PrEP than former LAI-CAB users reinitiate it.

## Discussion

In this cohort study, LAI-CAB users accounted for 3% of all US PrEP users from 2022 to 2024. The growth of LAI-CAB use was modest, with an average biannual increase of 3170 users. Overall, in their first year of LAI-CAB care, one-half of users were LAI-CAB persistent, and after accounting for modality switch, more than one-half were PrEP persistent (57%). Over a 2-year period, fewer than one-quarter (23%) were LAI-CAB persistent and fewer than one-third were PrEP persistent (30%).

LAI-CAB uptake was low compared with oral PrEP. A higher proportion of LAI-CAB users (26%) were covered by Medicaid than were oral PrEP users (14%), possibly indicating lower access barriers for Medicaid recipients compared with users with other insurance types. Conversely, a lower proportion of LAI-CAB users (2%) than of oral PrEP users (15%) were enrolled in assistance programs, suggesting potential challenges in accessing LAI-CAB through this channel. Further exploration of facilitators and barriers in insurance coverage could provide insights into optimizing LAI-CAB access across insurance types. We observed slightly higher relative levels of LAI-CAB use (3551 users [15%]) than oral PrEP use (83 274 users [11%]) use among female users, which might reflect greater familiarity with injectable medications, such as contraceptives, or increased acceptability of injectable PrEP formulations.^25^ Black and Hispanic individuals had 1.21 and 1.16 times the odds of LAI-CAB (vs oral PrEP) use, respectively, compared with White individuals. This may indicate greater acceptability of LAI-CAB among Black and Hispanic populations, which is particularly important given documented PrEP use inequities with oral PrEP.^26^

To our knowledge, this study is the first to document 2-year persistence for LAI-CAB users nationally. Persistence in oral PrEP care has proven to be challenging.^27,28^ In modeling studies, low levels of persistence led to limited progress toward ending the HIV epidemic.^29,30^ In our analysis, first-year LAI-CAB persistence (50%) was lower than previous national analyses of oral PrEP persistence (56%),^31^ and over a 2-year period, LAI-CAB persistence (23%) was substantially lower than oral PrEP persistence (41%).^31^ After accounting for modality switch, PrEP persistence (57%) was comparable to oral PrEP persistence at year 1 but lower at year 2 (30%). These findings suggest that there could be additional persistence barriers unique to LAI-CAB.

Clinical trials reported high LAI-CAB persistence at year 1 (73%-90%)^9,10,32^ and year 2 (85%).^10^ Similar levels were reported from single clinic^33,34^ and cohort observational studies,^35,36^ with 12-month persistence ranging from 75% to 90%.^33,34,35,36^ Other single clinic observational studies with shorter follow-up (6 months) also reported high persistence of 72%^37^ and 83%.^38^ Overall LAI-CAB persistence observed in our national analysis was lower than levels reported in these and other small sample size studies, even after accounting for modality switch. In comparison, our data include most LAI-CAB prescriptions in the US, reflecting clinical practice conditions of PrEP provision. Outside of research studies and in more diverse clinical settings, there may be limitations to staff time for encouraging persistence. Persistence estimates across studies should be interpreted with caution because of varying assessment periods, with shorter follow-up periods potentially creating a false impression of higher persistence by failing to capture subsequent discontinuation. The success demonstrated by clinics with high 12-month LAI-CAB persistence^33,34^ demonstrates that such outcomes are achievable with the right strategies and adequate resources; studies are needed to identify strategies implemented by these clinics to allow their practices to be modeled or adapted by other clinics to improve persistence. It remains unclear whether biannual LAI-PrEP will face similar hurdles observed with oral and bimonthly modalities, but clinics implementing biannual LAI-PrEP can draw lessons from the challenges with persistence of other modalities. For individuals who switched to oral PrEP as observed in our study, although the factors driving this transition remain unclear, the switch may be influenced by individual-level (eg, patient preference) or systemic-level (eg, insurance) factors. As more PrEP options become available, persistence assessment should account for switching between available modalities to better reflect actual patient engagement in PrEP care. Higher persistence among older adults, previously observed with oral PrEP,^31^ is also reflected in LAI-CAB care, suggesting that older adults have higher persistence across PrEP modalities. In our analyses, female users had lower persistence than male users across both 1-year and 2-year follow-up periods. Although there were slightly more female users initiating LAI-CAB than oral PrEP, it did not translate into better LAI-CAB persistence. Although Black and Hispanic individuals were more likely to initiate LAI-CAB compared with White individuals, persistence was not meaningfully different across racial and ethnic subgroups, suggesting that individuals of different races and ethnicities may be facing common persistence barriers. Collectively, these findings highlight the need to better understand barriers to LAI-CAB and PrEP persistence, including both individual-level and system-level factors that can have complex interactions; for instance, billing challenges can interact with patient-level factors such as insurance change or geographic relocation.^39^

From 2022 to 2024, LAI-CAB remained a small fraction of overall PrEP use in the US; most people used oral PrEP. Both before and after the Food and Drug Administration’s approval of LAI-CAB in December 2021, it was described as a game-changer in HIV prevention, eliminating the need for daily pills, providing an alternative for individuals facing challenges with pill taking, and reducing privacy and stigma concerns associated with taking oral medication.^4,5,6,7,8,40,41^ Numerous preference and acceptability studies reported high (>50%) stated preference for LAI-CAB among PrEP-eligible populations.^11,12,13,14,15^ Despite stated preferences, LAI-CAB accounted for only 3% of overall PrEP use in the US from 2022 to 2024, highlighting a substantial gap between reported interest and actual uptake, which may be explained in part by a combination of individual and structural barriers, such as insurance requirements, medication acquisition, and drug safety concerns.^18,40,42^ Lenacapavir, like CAB, has been regarded as a potential game-changer in HIV prevention.^43^ Although uptake trends for lenacapavir are not yet known and it is too early to characterize potential barriers to uptake, our study provides insights into clinical practice performance of LAI-CAB, in particular demonstrating problems with persistence in care. Although further research is needed to identify barriers to LAI-CAB implementation and sustained use, if persistence challenges are inherent to the health care system rather than to dosing intervals, injectable lenacapavir’s general population implementation and scale-up may face similar constraints. The public health system should seek to minimize barriers to both initiating and persisting in injectable PrEP care.

### Limitations

This study has some limitations. First, 48% of race and ethnicity data were missing; this was addressed using hot deck imputation, although such high levels of missingness still pose challenges to data interpretation. Second, we used insurance and copayment data from a user’s first PrEP claim, an approach that does not account for potential changes in patients’ insurance types or copayments over time. Third, claims datasets are inherently limited in the extent of available covariates; for instance, this dataset does not contain gender identity or sexual risk behavior information. The absence of such contextual information limits examination of LAI-CAB initiation and persistence outcomes by such key characteristics.

## Conclusions

Using national PrEP claims data to examine LAI-CAB user growth and persistence in care, LAI-CAB uptake was modest, representing a small portion (4% in the second biannual period of 2024) of overall US PrEP use. Persistence in LAI-CAB was about half in year 1, and after 2 years, about one-quarter. The implementation of more effective new PrEP modalities alone is unlikely to substantially increase PrEP use in the US, and future research should examine structural supports and behavioral interventions to facilitate scale-up of new, highly efficacious modalities.

## Notes

Supplement 1. eTable 1. LAI-CAB and PrEP persistence (assessed triannually) stratified by sociodemographic variables in the US, 2022-2024, among persons with at least 1 year of follow-up (n=12,118) and at least 2 years of follow-up (n=3,381) eTable 2. LAI-CAB and PrEP persistence (assessed annually) stratified by sociodemographic variables in the US, 2022-2024, among persons with at least 1 year of follow-up (n=12,118) and at least 2 years of follow-up (n=3,381) eTable 3. Sociodemographic Characteristics of PrEP users in the US, 2022-2024 (n=781,040) and Multivariable Logistic Regression of LAI-CAB use eTable 4. Sociodemographic characteristics of PrEP users in the US, 2022-2024 (n=781,040) and Firth penalized multivariable logistic regression of LAI-PrEP use eTable 5. Sociodemographic characteristics of PrEP users in the US, 2022-2024 (n=781,040) and multivariable logistic regression of LAI-PrEP use eTable 6. Unadjusted and Adjusted Odds Ratios of Multivariable Logistic Regression of ever LAI-CAB use versus Oral PrEP use only

Supplement 2. Data Sharing Statement
