# Supplementary material for: Long-Acting Injectable Cabotegravir Use and Persistence Over 2 Years
Source: JAMA Netw Open. 2026 Jun 26;9(6):e2620699. doi: 10.1001/jamanetworkopen.2026.20699 (PMC13309869; doi:10.1001/jamanetworkopen.2026.20699)
Supplement: Supplement 1. — eTable 1. LAI-CAB and PrEP persistence (assessed triannually) stratified by sociodemographic variables in the US, 2022-2024, among persons with at least 1 year of follow-up (n=12,118) and at least 2 years of follow-up (n=3,381) eTable 2. LAI-CAB and PrEP persistence (assessed annually) stratified by sociodemographic variables in the US, 2022-2024, among persons with at least 1 year of follow-up (n=12,118) and at least 2 years of follow-up (n=3,381) eTable 3. Sociodemographic Characteristics of PrEP users in the US, 2022-2024 (n=781,040) and Multivariable Logistic Regression of LAI-CAB use eTable 4. Sociodemographic characteristics of PrEP users in the US, 2022-2024 (n=781,040) and Firth penalized multivariable logistic regression of LAI-PrEP use eTable 5. Sociodemographic characteristics of PrEP users in the US, 2022-2024 (n=781,040) and multivariable logistic regression of LAI-PrEP use eTable 6. Unadjusted and Adjusted Odds Ratios of Multivariable Logistic Regression of ever LAI-CAB use versus Oral PrEP use only [file jamanetwopen-e2620699-s001.pdf]

Supplemental Online Content

Koh SHE, Huang W, Hall EW, et al. Long -acting injectable cabotegravir use and persistence over 2 years. *JAMA New Open*. 2026;9(6):e2620699. doi:10.1001/jamanetworkopen.2026.20699

eTable 1. LAI-CAB and PrEP persistence (assessed triannually) stratified by sociodemographic variables in the US, 2022-2024, among persons with at least 1 year of follow-up (n=12,118) and at least 2 years of follow-up (n=3,381)

eTable 2. LAI-CAB and PrEP persistence (assessed annually) stratified by sociodemographic variables in the US, 2022-2024, among persons with at least 1 year of follow-up (n=12,118) and at least 2 years of follow-up (n=3,381)

eTable 3. Sociodemographic Characteristics of PrEP users in the US, 2022-2024 (n=781,040) and Multivariable Logistic Regression of LAI-CAB use

eTable 4. Sociodemographic characteristics of PrEP users in the US, 2022-2024 (n=781,040) and Firth penalized multivariable logistic regression of LAI-PrEP use

eTable 5. Sociodemographic characteristics of PrEP users in the US, 2022-2024 (n=781,040) and multivariable logistic regression of LAI-PrEP use

eTable 6. Unadjusted and Adjusted Odds Ratios of Multivariable Logistic Regression of ever LAI-CAB use versus Oral PrEP use only

This supplemental material has been provided by the authors to give readers additional information about their work.

© 2026 Koh SHE et al. *JAMA Network Open*.

Supplement Materials

Sensitivity Analysis of Long-acting injectable Cabotegravir (LAI-CAB) Persistence Measure

eTable 1 shows LAI-CAB and PrEP persistence assessed using a triannual (4-monthly) definition, whereas **eTable 2** shows LAI-CAB and PrEP persistence assessed using an annual (12 monthly) definition.

eTable 1: LAI-CAB and PrEP persistence (assessed triannually) stratified by sociodemographic variables in the US, 2022-2024, among persons with at least 1 year of follow-up (n=12,118) and at least 2 years of follow-up (n=3,381)

	**Persons with at least 1 year of follow-up**					**Persons with at least 2 years of follow-up**												
	**LAI-CAB Initiation**	**LAI-CAB persistence**		**PrEP persistence**		**LAI-CAB Initiation**	**LAI-CAB persistence**						**PrEP persistence**					
**Variables**		**Year 1**		**Year 1**			**Year 1**		**Year 2[2]**		**Year 1-2[3]**		**Year 1**		**Year 2[2]**		**Year 1-2[3]**	
	**n**	**n**	**%**	**n**	**%**	**n**	**n**	**%**	**n**	**%**	**n**	**%**	**n**	**%**	**n**	**%**	**n**	**%**
Overall	12,118	6,129	51%	6,974	58%	3,381	1,621	48%	930	28%	825	24%	1,914	57%	1,236	37%	1,079	32%
Age (Years)																		
<= 18	87	27	31%	28	32%	35	12	34%	3	9%[a]	3	9%[a]	13	37%	4	11%[a]	4	11%[a]
19 – 30	3,854	1,758	46%	1,990	52%	1,092	474	43%	238	22%	210	19%	561	51%	317	29%	270	25%
31 – 40	4,358	2,210	51%	2,530	58%	1,150	567	49%	328	29%	281	24%	671	58%	438	38%	376	33%
41 – 50	2,123	1,134	53%	1,307	62%	580	273	47%	166	29%	148	26%	332	57%	232	40%	200	34%
> 50	1,696	1,000	59%	1,119	66%	524	295	56%	195	37%	183	35%	337	65%	245	47%	229	44%
Sex[1]																		
Male	10,462	5,543	53%	6,336	61%	2,919	1,472	50%	864	30%	765	26%	1,749	60%	1,160	40%	1,010	35%
Female	1,655	586	35%	638	39%	462	149	32%	66	14%	60	13%	165	36%	76	16%	69	15%
Race and ethnicity[b]																		

© 2026 Koh SHE et al. *JAMA Network Open*.

Asian	334	164	49%	193	58%	96	51	53%	22	23%	20	21%	59	61%	33	34%	29	30%
Black / African American	2,292	1,076	47%	1,215	53%	619	280	45%	157	25%	138	22%	316	51%	202	33%	171	28%
Hispanic	2,245	1,141	51%	1,305	58%	661	331	50%	174	26%	156	24%	385	58%	239	36%	206	31%
White / Caucasian	6,749	3,521	52%	4,005	59%	1872	913	49%	553	30%	491	26%	1,103	59%	729	39%	647	35%
Others	219	110	50%	125	57%	70	23	33%	13	19%	11	16%[a]	28	40%	22	31%	17	24%
<u>Payer Type[1]</u>																		
Commercial	8,402	4,509	54%	5,149	61%	2,271	1,126	50%	664	29%	576	25%	1,355	60%	907	40%	778	34%
Government	14	7	50%[a]	7	50%[a]	1	0	0%	0	0%	0	0%	0	0%	0	0%	0	0%
Medicare	408	213	52%	241	59%	133	67	50%	44	33%	43	32%	78	59%	50	38%	48	36%
Medicaid	3,174	1,333	42%	1,497	47%	955	416	44%	213	22%	197	21%	464	49%	267	28%	241	25%
Assistance Programs	71	45	63%	52	73%	14	8	57%[a]	6	43%[a]	6	43%[a]	11	79%[a]	9	64%[a]	9	64%[a]
Cash	38	16	42%	20	53%	2	1	50%[a]	1	50%[a]	1	50%[a]	2	100%[a]	1	50%[a]	1	50%[a]
<u>Co-pay amount in USD[1]</u>																		
0	6,612	3,394	51%	3,840	58%	1,856	912	49%	513	28%	469	25%	1,059	57%	671	36%	605	33%
1 - 10	281	109	39%	133	47%	130	40	31%	26	20%	25	19%	52	40%	31	24%	29	22%
11 - 100	496	285	57%	328	66%	148	66	45%	45	30%	40	27%	91	61%	63	43%	57	39%
101 - 500	239	135	56%	162	68%	96	46	48%	45	47%	34	35%	60	63%	54	56%	42	44%
501 - 1,000	77	49	64%	55	71%	28	16	57%	14	50%	11	39%[a]	19	68%	18	64%	13	46%
> 1,000	249	141	57%	160	64%	83	35	42%	24	29%	21	25%	41	49%	32	39%	25	30%

[1] Values do not add up due to missing information. Missing race and ethnicity n = 279, payer type = 11, co-pay = 4,164
[2] Year two persistence was assessed regardless of persistence status in Year 1.
[3] Persistent in all six triannual periods.
[a] Percentages generated from a numerator of less than 12 are considered unstable and should be interpreted with caution (adapted from AIDSVu[23]).
[b] Hot deck imputed.

© 2026 Koh SHE et al. *JAMA Network Open*.

LAI-CAB persistence was defined as having at least one claim for cabotegravir in each triannual period (4-month interval). PrEP persistence was defined as having at least one claim for any PrEP drugs i.e., cabotegravir, TDF/FTC and TAF/FTC in each triannual period, to account for those who switch from LAI-CAB to oral PrEP. Users were followed from initiation of LAI-CAB to last available data point in the dataset (December 31, 2024). Users with at least one year of follow-up from LAI-CAB initiation were included in the assessment of year one persistence. Among users with at least one year of follow up, users with at least two years of follow-up from LAI-CAB initiation were included in the two-year persistence analysis, and only users who were persistent in year one was evaluated for continued persistence in year two. The denominators (n) of the respective subgroups are shown in the "Initiation" columns and percentages represent the proportion of users who were persistent.

eTable 2: LAI-CAB and PrEP persistence (assessed annually) stratified by sociodemographic variables in the US, 2022-2024, among persons with at least 1 year of follow-up (n=12,118) and at least 2 years of follow-up (n=3,381)

	Persons with at least 1 year of follow-up					Persons with at least 2 years of follow-up													
	LAI-CAB Initiation	LAI-CAB persistence		PrEP persistence		LAI-CAB Initiation	LAI-CAB persistence						PrEP persistence					
Variables		Year 1		Year 1			Year 1		Year 2[2]		Year 1-2[3]		Year 1		Year 2[2]		Year 1-2[3]	
	n	n	%	n	%	n	n	%	n	%	n	%	n	%	n	%	n	%
Overall	12,118	5103	42%	6,024	50%	3,381	1,384	41%	852	25%	693	20%	1,704	50%	1,165	34%	947	28%
Age (Years)																		
<= 18	87	23	26%	24	28%	35	12	34%	3	9%[a]	3	9%[a]	13	37%	4	11%[a]	4	11%[a]
19 – 30	3,854	1434	37%	1,690	44%	1,092	395	36%	216	20%	172	16%	486	45%	306	28%	236	22%
31 – 40	4,358	1838	42%	2,184	50%	1,150	488	42%	293	25%	233	20%	599	52%	410	36%	337	29%
41 – 50	2,123	955	45%	1,140	54%	580	227	39%	160	28%	134	23%	296	51%	218	38%	176	30%
> 50	1,696	853	50%	986	58%	524	262	50%	180	34%	151	29%	310	59%	227	43%	194	37%
Sex[1]																		
Male	10,462	4646	44%	5,518	53%	2,919	1,259	43%	788	27%	641	22%	1,566	54%	1,090	37%	887	30%
Female	1,655	457	28%	506	31%	462	125	27%	64	14%	52	11%	138	30%	75	16%	60	13%
Race and ethnicity[b]																		
Asian	334	135	40%	162	49%	96	40	42%	19	19%	16	17%	49	51%	29	30%	21	22%
Black / African American	2,292	900	39%	1,049	45%	619	243	39%	147	24%	120	19%	287	46%	193	31%	155	25%
Hispanic	2,245	931	41%	1,128	50%	661	272	41%	163	25%	134	20%	338	51%	232	35%	191	29%

© 2026 Koh SHE et al. *JAMA Network Open*.

White / Caucasian	6,749	2946	44%	3,469	51%	1872	786	42%	500	27%	402	21%	983	52%	682	36%	555	25%
Others	219	97	44%	109	50%	70	22	31%	13	19%[a]	12	17%[a]	26	37%	19	27%	16	23%
Payer Type[1]																		
Commercial	8,402	3759	45%	4,449	53%	2,271	966	43%	614	27%	488	21%	1,208	53%	865	38%	691	30%
Government	14	5	36%	5	36%[a]	1	0	0%	0	0%	0	0%	0	0%	0	0%	0	0%
Medicare	408	171	42%	206	50%	133	59	44%	40	30%	36	27%	71	53%	49	37%	44	33%
Medicaid	3,174	1110	35%	1,294	41%	955	347	36%	188	20%	160	17%	408	43%	237	25%	199	21%
Assistance Programs	71	40	56%	47	66%	14	8	57%[a]	6	43%[a]	6	43%[a]	11	79%[a]	9	64%[a]	9	64%[a]
Cash	38	14	37%	18	47%	2	1	50%[a]	1	50%[a]	1	50%[a]	2	100%[a]	2	100%[a]	2	100%[a]
Co-pay amount in USD[1]																		
0	6,612	2863	43%	3,377	51%	1,856	775	42%	452	24%	380	20%	944	51%	611	33%	515	28%
1 - 10	281	88	31%	115	41%	130	35	27%	27	21%	23	18%	47	36%	34	26%	29	22%
11 - 100	496	256	52%	298	60%	148	59	40%	45	30%	36	24%	81	55%	59	40%	49	33%
101 - 500	239	122	51%	146	61%	96	43	45%	41	43%	30	31%	57	59%	51	53%	39	41%
501 - 1,000	77	42	55%	47	61%	28	16	57%	13	46%	11	39%[a]	18	64%	15	54%	11	39%
> 1,000	249	126	51%	142	57%	83	35	42%	25	30%	21	25%	39	47%	31	37%	25	30%

[1] Values do not add up due to missing information. Missing race and ethnicity n = 279, payer type = 11, co-pay = 4,164
[2] Year two persistence was assessed regardless of persistence status in Year 1.
[3] Persistent in both years.
[a] Percentages generated from a numerator of less than 12 are considered unstable and should be interpreted with caution (adapted from AIDSVu[23]).
[b] Hot deck imputed.

LAI-CAB persistence was defined as having at least six claims for cabotegravir in year one and at least five claims for cabotegravir in year two. PrEP persistence was defined as having at least six claims for any PrEP drugs i.e., cabotegravir, TDF/FTC and TAF/FTC in year one and five claims for any PrEP drugs in year two, to account for those who switch from LAI-CAB to oral PrEP. Users were followed from initiation of LAI-CAB to last available data point in the dataset (December 31, 2024). Users with at least one year of follow-up from LAI-CAB initiation were included in the assessment of year one persistence. Among users with at least one year of follow up, users with at least two years of follow-up from LAI-CAB initiation were included in the two-year persistence analysis, and only users who were persistent in year one was evaluated for continued persistence in year two. The denominators (n) of the respective subgroups are shown in the “Initiation” columns and percentages represent the proportion of users who were persistent.

© 2026 Koh SHE et al. *JAMA Network Open*.

Missing Indicator Method

Missing race/ethnicity data was handled using the missing indicator approach. See **eTable 3** for adjusted odds ratios. Model discrimination was modest (c=0.63), the Hosmer-Lemeshow test indicated suboptimal fit (χ^2=54.4, p<0.001) and there was no evidence of collinearity.

eTable 3: Sociodemographic Characteristics of PrEP users in the US, 2022-2024 (n=781,040) and Multivariable Logistic Regression of LAI-CAB use

Variable	Overall PrEP Users[1]		Oral PrEP Users[1]		LAI-CAB Users[1]		Ever LAI-CAB vs Oral PrEP only
	n	%	n	%	n	%	aOR (95% CI)
Total	781,040	-	770,833	-	24,194	-	735,228
Age (year, mean ± SD)	36.4 ± 11.9	-	36.4 ± 11.9	-	37.1 ± 11.4	-	-
≤ 18	6,983	1%	6,854	1%	175	1%	0.71 (0.62, 0.83)
19 - 30	283,110	36%	279,749	36%	7,531	31%	0.83 (0.80, 0.86)
31 - 40	256,825	33%	253,274	33%	8,828	36%	Ref
41 - 50	120,211	15%	118,474	15%	4,281	18%	1.00 (0.96, 1.05)
> 50	113,911	15%	112,482	15%	3,379	14%	0.82 (0.77, 0.85)
Sex at birth[2]							
Male	695,388	89%	687,541	89%	20,642	85%	Ref
Female	85,633	11%	83,274	11%	3,551	15%	1.10 (1.05, 1.14)
Race and Ethnicity							
Asian	13,197	2%	13,105	2%	299	1%	0.82 (0.72, 0.94)
Black/African American	65,564	8%	64,394	8%	2,699	11%	1.39 (1.32, 1.47)
Hispanic	68,640	9%	67,724	9%	2,532	10%	1.33 (1.26, 1.40)
White/Caucasian	252,370	32%	250,073	32%	6,868	28%	Ref
Others	7,280	1%	7,208	1%	217	1%	1.02 (0.88, 1.19)
Missing	373,989	48%	368,329	48%	11,579	48%	1.07 (1.03, 1.11)
US Census Region[2]							
Northeast	176,024	23%	173,405	23%	6,003	25%	1.16 (1.12, 1.21)
Midwest	123,936	16%	122,512	16%	3,640	15%	1.03 (0.99, 1.08)
South	288,313	37%	284,997	37%	8,347	35%	Ref
West	174,382	22%	171,867	22%	5,610	23%	1.06 (1.02, 1.11)
Territories	1,864	<1%	1,836	<1%	57	<1%	1.12 (0.83, 1.51)
Payer Type[2]							
Commercial	520,012	67%	513,746	67%	16,334	68%	Ref
Government	14	<1%	3	<1%	19	<1%	-
Medicare	21,590	3%	21,220	3%	851	4%	1.67 (1.54, 1.81)
Medicaid	111,929	14%	108,589	14%	6,256	26%	2.47 (2.39, 2.56)
Assistance Programs	118,658	15%	118,534	15%	509	2%	0.68 (0.65, 0.72)
Cash	3,369	<1%	3,278	<1%	207	1%	0.49 (0.31, 0.80)
Co-pay amount in USD[2]							
0	625,519	83%	619,959	83%	13,507	82%	Ref
1 - 10	33,243	4%	33,024	4%	477	3%	0.75 (0.70, 0.81)
11 - 100	69,395	9%	69,120	9%	1,040	6%	1.05 (0.99, 1.10)
101 - 500	14,991	2%	14,836	2%	471	3%	1.43 (1.30, 1.57)
501 - 1,000	6,246	1%	6,184	1%	181	1%	1.44 (1.25, 1.66)

© 2026 Koh SHE et al. *JAMA Network Open*.

> 1,000	7,089	1%	6,841	1%	705	4%	2.61 (2.35, 2.90)

[1] Values from Oral PrEP and LAI-CAB users columns do not add up to PrEP users column due to persons who used more than one type of PrEP medication from 2022 to 2024.
[2] Values do not add up due to missing information. Sex missing, n (%) = 19 (<1%); US census region missing, n (%) = 16,521 (2%); Payer type missing, n (%) = 5468 (<1%); co-pay amount, n (%) 24,557 (3%).

Firth Penalized Multivariable Logistic Regression

Adjusted odds ratios (aOR) were stable across modeling approaches, and Firth's penalized logistic regression (c=0.63) did not meaningfully change the estimates because of population size imbalance between LAI-PrEP and oral PrEP users (**eTable 4**). The Hosmer-Lemeshow test indicated suboptimal fit ($\chi^2 = 54.6$, $p < 0.001$).

eTable 4: Sociodemographic characteristics of PrEP users in the US, 2022-2024 (n=781,040) and Firth penalized multivariable logistic regression of LAI-PrEP use

Variable	Overall PrEP Users[1]		Oral PrEP Users[1]		LAI-PrEP Users[1]		Ever LAI-PrEP vs Oral PrEP only
	n	%	n	%	n	%	aOR (95% CI)
Total	781,040	-	770,833	-	24,194	-	735,228
Age (year, mean ± SD)	36.4 ± 11.9	-	36.4 ± 11.9	-	37.1 ± 11.4	-	-
≤ 18	6,983	1%	6,854	1%	175	1%	0.72 (0.62, 0.83)
19 - 30	283,110	36%	279,749	36%	7,531	31%	0.83 (0.80, 0.86)
31 - 40	256,825	33%	253,274	33%	8,828	36%	Ref
41 - 50	120,211	15%	118,474	15%	4,281	18%	1.00 (0.96, 1.05)
> 50	113,911	15%	112,482	15%	3,379	14%	0.82 (0.77, 0.85)
Sex at birth[2]							
Male	695,388	89%	687,541	89%	20,642	85%	Ref
Female	85,633	11%	83,274	11%	3,551	15%	1.10 (1.05, 1.14)
Race and Ethnicity							
Asian	13,197	2%	13,105	2%	299	1%	0.82 (0.73, 0.94)
Black/African American	65,564	8%	64,394	8%	2,699	11%	1.39 (1.32, 1.47)
Hispanic	68,640	9%	67,724	9%	2,532	10%	1.33 (1.26, 1.40)
White/Caucasian	252,370	32%	250,073	32%	6,868	28%	Ref
Others	7,280	1%	7,208	1%	217	1%	1.03 (0.88, 1.19)
Missing	373,989	48%	368,329	48%	11,579	48%	1.07 (1.03, 1.11)
US Census Region[2]							
Northeast	176,024	23%	173,405	23%	6,003	25%	1.16 (1.12, 1.21)
Midwest	123,936	16%	122,512	16%	3,640	15%	1.03 (0.99, 1.08)
South	288,313	37%	284,997	37%	8,347	35%	Ref
West	174,382	22%	171,867	22%	5,610	23%	1.06 (1.02, 1.11)
Territories	1,864	<1%	1,836	<1%	57	<1%	1.13 (0.84, 1.52)
Payer Type[2]							
Commercial	520,012	67%	513,746	67%	16,334	68%	Ref
Government	14	<1%	3	<1%	19	<1%	-
Medicare	21,590	3%	21,220	3%	851	4%	1.67 (1.54, 1.81)
Medicaid	111,929	14%	108,589	14%	6,256	26%	2.47 (2.39, 2.56)
Assistance Programs	118,658	15%	118,534	15%	509	2%	0.68 (0.65, 0.72)
Cash	3,369	<1%	3,278	<1%	207	1%	0.51 (0.32, 0.81)

© 2026 Koh SHE et al. *JAMA Network Open*.

Co-pay amount in USD[2]							
0	625,519	83%	619,959	83%	13,507	82%	Ref
1 - 10	33,243	4%	33,024	4%	477	3%	0.75 (0.70, 0.81)
11 - 100	69,395	9%	69,120	9%	1,040	6%	1.05 (0.99, 1.10)
101 - 500	14,991	2%	14,836	2%	471	3%	1.43 (1.30, 1.57)
501 - 1,000	6,246	1%	6,184	1%	181	1%	1.44 (1.25, 1.66)
> 1,000	7,089	1%	6,841	1%	705	4%	2.61 (2.35, 2.90)

[1] Values from Oral PrEP and LAI-PrEP users columns do not add up to PrEP users column due to persons who used more than one type of PrEP medication from 2022 to 2024.
[2] Values do not add up due to missing information. Sex missing, n (%) = 19 (<1%); US census region missing, n (%) = 16,521 (2%); Payer type missing, n (%) = 5468 (<1%); co-pay amount, n (%) 24,557 (3%).

Multivariable Logistic Regression Excluding Missing Category of Race and Ethnicity

eTable 5 shows the adjusted odds ratios (aOR) for each of the covariates after excluding users with missing Race and Ethnicity information from the multivariable logistic regression model, which did not meaningfully change the estimates (c=0.61). The Hosmer-Lemeshow test indicated suboptimal fit ($\chi^2 = 32.8$, $p < 0.001$).

eTable 5: Sociodemographic characteristics of PrEP users in the US, 2022-2024 (n=781,040) and multivariable logistic regression of LAI-PrEP use

**Variable**	**Overall PrEP Users[1]**		**Oral PrEP Users[1]**		**LAI-PrEP Users[1]**		**Ever LAI-PrEP vs Oral PrEP only**
	**n**	**%**	**n**	**%**	**n**	**%**	**aOR (95% CI)**
Total	781,040	-	770,833	-	24,194	-	389,880
Age (year, mean ± SD)	36.4 ± 11.9	-	36.4 ± 11.9	-	37.1 ± 11.4	-	-
≤ 18	6,983	1%	6,854	1%	175	1%	0.64 (0.26, 1.56)
19 - 30	283,110	36%	279,749	36%	7,531	31%	0.85 (0.81, 0.89)
31 - 40	256,825	33%	253,274	33%	8,828	36%	Ref
41 - 50	120,211	15%	118,474	15%	4,281	18%	0.99 (0.94, 1.05)
> 50	113,911	15%	112,482	15%	3,379	14%	0.85 (0.81, 0.91)
Sex at birth[2]							
Male	695,388	89%	687,541	89%	20,642	85%	Ref
Female	85,633	11%	83,274	11%	3,551	15%	0.99 (0.93, 1.06)
Race and Ethnicity							
Asian	13,197	2%	13,105	2%	299	1%	0.83 (0.73, 0.94)
Black/African American	65,564	8%	64,394	8%	2,699	11%	1.44 (1.37, 1.52)
Hispanic	68,640	9%	67,724	9%	2,532	10%	1.35 (1.28, 1.42)
White/Caucasian	252,370	32%	250,073	32%	6,868	28%	Ref
Others	7,280	1%	7,208	1%	217	1%	1.03 (0.89, 1.20)
Missing	373,989	48%	368,329	48%	11,579	48%	-
US Census Region[2]							
Northeast	176,024	23%	173,405	23%	6,003	25%	1.16 (1.11, 1.22)
Midwest	123,936	16%	122,512	16%	3,640	15%	1.05 (0.99, 1.11)
South	288,313	37%	284,997	37%	8,347	35%	Ref
West	174,382	22%	171,867	22%	5,610	23%	1.05 (0.99, 1.11)
Territories	1,864	<1%	1,836	<1%	57	<1%	-

© 2026 Koh SHE et al. *JAMA Network Open*.

Payer Type[2]							
Commercial	520,012	67%	513,746	67%	16,334	68%	Ref
Government	14	<1%	3	<1%	19	<1%	-
Medicare	21,590	3%	21,220	3%	851	4%	1.56 (1.41, 1.72)
Medicaid	111,929	14%	108,589	14%	6,256	26%	2.17 (2.06, 2.28)
Assistance Programs	118,658	15%	118,534	15%	509	2%	0.76 (0.71, 0.81)
Cash	3,369	<1%	3,278	<1%	207	1%	0.68 (0.36, 1.27)
Co-pay amount in USD[2]							
0	625,519	83%	619,959	83%	13,507	82%	Ref
1 - 10	33,243	4%	33,024	4%	477	3%	0.81 (0.73, 0.89)
11 - 100	69,395	9%	69,120	9%	1,040	6%	1.04 (0.97, 1.11)
101 - 500	14,991	2%	14,836	2%	471	3%	1.37 (1.21, 1.55)
501 - 1,000	6,246	1%	6,184	1%	181	1%	1.53 (1.29, 1.83)
> 1,000	7,089	1%	6,841	1%	705	4%	2.34 (2.03, 2.70)

[1] Values from Oral PrEP and LAI-PrEP users columns do not add up to PrEP users column due to persons who used more than one type of PrEP medication from 2022 to 2024.
[2] Values do not add up due to missing information. Sex missing, n (%) = 19 (<1%); US census region missing, n (%) = 16,521 (2%); Payer type missing, n (%) = 5468 (<1%); co-pay amount, n (%) 24,557 (3%).

© 2026 Koh SHE et al. *JAMA Network Open*.

Unadjusted Odds Ratios of Multivariable Logistic Regression

eTable 6 shows the unadjusted and adjusted odds ratios of the multivariable logistic regression of LAI-CAB use versus Oral PrEP use only.

eTable 6: Unadjusted and Adjusted Odds Ratios of Multivariable Logistic Regression of ever LAI-CAB use versus Oral PrEP use only

Variable	Ever LAI-CAB vs Oral PrEP only	
	**Unadjusted OR (95% CI)**	**Adjusted OR (95% CI)**
Age (year, mean ± SD)	-	-
≤ 18	0.91 (0.80, 1.05)	0.70 (0.60, 0.81)
19 - 30	0.84 (0.82, 0.87)	0.83 (0.80, 0.86)
31 - 40	Ref	Ref
41 - 50	1.01 (0.97, 1.05)	1.00 (0.96, 1.05)
> 50	0.84 (0.80, 0.87)	0.81 (0.78, 0.85)
Sex at birth[2]		
Male	Ref	Ref
Female	1.41 (1.36, 1.47)	1.10 (1.06, 1.15)
Race and Ethnicity[3]		
Asian	0.91 (0.84, 0.98)	0.90 (0.83, 1.11)
Black/African American	1.28 (1.24, 1.33)	1.21 (1.17, 1.26)
Hispanic	1.18 (1.14, 1.22)	1.16 (1.12, 1.21)
White/Caucasian	Ref	Ref
Others	1.05 (0.95, 1.16)	1.00 (0.89, 1.11)
US Census Region[2]		
Northeast	1.18 (1.15, 1.23)	1.15 (1.11, 1.19)
Midwest	1.02 (0.98, 1.06)	1.01 (0.97, 1.06)
South	Ref	Ref
West	1.12 (1.08, 1.15)	1.04 (1.00, 1.08)
Territories	1.06 (0.81, 1.38)	1.08 (0.80, 1.46)
Payer Type[2]		
Commercial	Ref	Ref
Government	-	-
Medicare	1.29 (1.29, 1.29)	1.68 (1.55, 1.82)
Medicaid	2.54 (2.53, 2.54)	2.50 (2.41, 2.59)
Assistance Programs	0.64 (0.63, 0.64)	0.68 (0.65, 0.72)
Cash	1.30 (1.30, 1.30)	0.49 (0.31, 0.80)
Co-pay amount in USD[2]		
0	Ref	Ref
1 - 10	0.98 (0.92, 1.05)	0.75 (0.70, 0.81)
11 - 100	0.84 (0.80, 0.89)	1.04 (0.99, 1.10)
101 - 500	1.20 (1.09, 1.31)	1.43 (1.30, 1.57)
501 - 1,000	1.27 (1.10, 1.45)	1.44 (1.25, 1.66)
> 1,000	2.13 (1.92, 2.36)	2.61 (2.35, 2.90)

© 2026 Koh SHE et al. *JAMA Network Open*.
