# Supplementary material for: Long-Acting Injectable Cabotegravir Use and Persistence Over 2 Years
Source: JAMA Netw Open. 2026 Jun 26;9(6):e2620699. doi: 10.1001/jamanetworkopen.2026.20699 (PMC13309869; doi:10.1001/jamanetworkopen.2026.20699)
Supplement: Supplement 2. — Data Sharing Statement [file jamanetwopen-e2620699-s002.pdf]

Data Sharing Statement

Koh. Long-Acting Injectable Cabotegravir Use and Persistence Over 2 Years. *JAMA Netw Open*. Published June 26, 2026. doi:10.1001/jamanetworkopen.2026.20699

Data
Data available: No

Additional Information
Explanation for why data not available: The data used for this study were purchased from a claims data aggregator, and the contract with the claims aggregator (which is standard for this type of data purchase) does not allow for us to make the dataset publicly available. The data are available, however, for purchase from the claims aggregator.
